# A Qualitative Study of Family Caregiver Experiences of Managing Incontinence in Stroke Survivors

**DOI:** 10.1371/journal.pone.0129540

**Published:** 2015-06-12

**Authors:** Chien-Ning Tseng, Guey-Shiun Huang, Po-Jui Yu, Meei-Fang Lou

**Affiliations:** 1 Department of Nursing, Cardinal Tien College of Healthcare and Management, New Taipei City, Taiwan; 2 School of Nursing, College of Medicine, National Taiwan University, Taipei, Taiwan; Örebro University, SWEDEN

## Abstract

**Background:**

Incontinence is a common problem faced by family caregivers that is recognized as a major burden and predictor of institutionalization. However, few studies have evaluated the experiences of family caregivers caring for stroke survivors with incontinence.

**Purpose:**

To describe experiences of caregivers managing incontinence in stroke survivors.

**Design:**

This qualitative descriptive study employed a grounded-theory approach.

**Methods:**

Semi-structured in-depth interviews with ten family caregivers of stroke survivors with incontinence were conducted during 2011. Audiotaped interviews were transcribed and analyzed using content analysis.

**Findings:**

Data analysis identified four themes: chaos, hypervigilance, exhaustion, and creating a new life. There were nine related subcategories: fluster, dirtiness, urgency, fear of potential health-hazard, physically demanding and time-consuming, mentally draining, financial burden, learning by doing, and attitude adjustment. Together, these described a process of struggling to cope with the care of stroke survivors with urinary/fecal incontinence. Of the four categories, “creating a new life” developed gradually over time to orient caregivers to their new life, while the other three categories occurred in a chronological order.

**Conclusion:**

The research highlighted unique caring experiences of family caregivers of stroke patients, which focused solely on the ‘incontinence issue’. Understanding these experiences may help nurses provide better support and resources for family caregivers when caring for stroke survivors with incontinence.

## Introduction

Stroke is a disabling illness that is prevalent in both developed and developing countries [1]. In Taiwan, stroke is the third leading cause of mortality [2]. This is a serious neurological event primarily affecting older people, and its consequences include mobility problems, cognitive impairment, urinary/fecal incontinence, speech and communication difficulties, and personality changes that often require lifelong assistance [3–4]. A large proportion of stroke survivors are therefore dependent on family caregivers who must assume multiple responsibilities, and this can lead to frustration, particularly with those tasks that are most time-consuming, such as managing incontinence [5]. An epidemiological survey of caregivers of stroke survivors reported that handling incontinence was the third most stress-inducing stroke-related patient problem after patient depression and patient loneliness [6]. However, handling bowel and bladder problems was the most stressful physical care activity reported.

Bowel and bladder control are associated with a person’s privacy, self-esteem, and dignity. Loss of bowel or bladder control is linked to feelings of personal incompetence, embarrassment, and shame, in addition to being a social stigma. Patients with incontinence require help from relatives, which impacts emotional, physical, and social well-being of both patients and caregivers [7–10]. Patients with incontinence require help with toileting or diaper changes several times per day, as well as at night, which can leave family caregivers with little time for themselves. Not only is this laborious work but caregivers also experience unpleasant odors and may experience disgust [10]. Consequently, incontinence is a significant precipitating factor when deciding to seek nursing home placements [11–12]. Quantitative research has shown that those caring for stroke survivors had higher levels of depression and psychological morbidity, and poorer quality of life than those caring for people with other chronic conditions [13–17].

The literature relating to the experiences of caring for stroke survivors is now relatively extensive. However, although several qualitative studies have focused on the issue of incontinence among non-stroke patients [5, 18–20], few have concentrated on stroke survivors with incontinence. Understanding the experiences of family caregivers of stroke survivors with incontinence would assist health personnel in better supporting the well-being of this vulnerable population. Therefore, this study aimed to describe the experiences of family caregivers when managing incontinence in stroke survivors.

## Methods

### Design

We used a qualitative design with a grounded theory method described by Corbin and Strauss [21]. The grounded theory approach was used to identify initial themes and categories that describe the experiences and human interactions underway when family caregivers are caring for stroke survivors with urinary/fecal incontinence.

### Participants and setting

Caregivers were purposefully selected from contacts obtained by outpatient staff at clinics of the National Taiwan University Hospital. Participants were included if they were adult family caregivers of stroke survivors who had cared for relatives with urinary, fecal, or dual incontinence for at least 1 month. Semi-structured, face-to-face, tape-recorded interviews were conducted with 10 adult family caregivers (two men and eight women) with home-based caregiver responsibilities. The characteristics of family caregivers and their relationship to the stroke survivors are presented in Table 1. The ages of the caregivers varied from 21 to 78 years, and they had different relationships to the incontinent patients: two granddaughters, one daughter-in-law, one son-in-law, three daughters, one son, and two women spouses. None were in paid employment.

**Table 1:** Characteristics of participants and stroke survivors.

Characteristics of		Characteristics of	
Family Caregivers	N	Stroke Survivors	N
**Gender**		**Gender**	
Male	2	Male	5
Female	8	Female	5
**Age (years)**		**Age (years)**	
20–40	4	65–74	4
40–64	3	75–84	4
65-and above	3	85-and above	2
**Type of Relationship**		**Duration of Stroke (months)**	
Wife/Husband	2	1–6 months	4
Daughter/Son	4	7–12 months	3
Daughter-in-low/Son-in-low	2	13-	3
Granddaughter/Grandson	2	**Barthel Index**	
**Education**		0–20 (total dependence)	9
Illiterate	1	61–90 (moderate dependence)	1
Elementary school	1	91–99 (slight dependence)	0
High school	2	100 (independence)	0
College and above	6	^**a**^ **NIHSS**	
**Employment**		0–3	0
Yes	0	4–15	3
No	10	>16	7
		**Incontinence**	
		Urinary	10
		Fecal	7
		Both	7
		**Indwelling urinary catheter**	
		**placement**	
		Yes	1
		No	9

^a^NIHSS: National Institutes of Health Stroke Scale.

The stroke survivors had a median age of 74.5 years (range, 68–86) and half were men. All stroke survivors had limitations in their activities of daily living (ADLs) with the majority being totally dependent (n = 9). Seven stroke survivors presented with an initial National Institutes of Health Stroke Scale (NIHSS) score ≥16 (i.e., severe stroke) and dual incontinence. The mean time since the stroke was 18.9 months (range, 1–63).

### Data collection process

Data were collected from April to August 2011. An interview guide was developed and then refined through discussion by the research team. The interview began with a broad central question regarding the caregiver’s experience: Tell me what it is like to care for a close relative who has incontinence. This question was discussed in all interviews and followed up by probing and follow-up questions in relation to this caring experience (for example, how do you deal with it?). The first author (CNT) carried out all the interviews. The interviews were conducted in a quiet room of the outpatient clinic and lasted 35 to 75 minutes. All the interviews were tape-recorded.

### Data analysis

The interview data were transcribed verbatim and transcripts were compared with audiotapes to verify accuracy. Following Corbin and Strauss’ guidelines, data collection and data analysis took place simultaneously [21]. Every interview was analyzed immediately afterwards in order to identify ideas, which provided the chance to compare material and to generate categories. The semantic contents were differentiated, abstracted and coded. Consistent with grounded theory, as the interviews progressed and analysis continued, constant comparative analysis was used to compare data and open coding determined core categories. Similar codes were grouped into categories to identify the main themes and subcategories.

The rigor of this qualitative study depended on the ability to capture and accurately represent the reality of the experience of caring for incontinence from the caregiver's perspective. Toward that end, a wider purposeful sampling allowed for interviews from of a broad spectrum of family caregivers, increasing the range of information and depth of knowledge. Obtaining data from different types of family members, rather than relying on a single type of relative, can provide a larger variety of caregiving experiences. Two investigators, CNT and MFL, reviewed the coding together to ensure that codes were data-driven. Differences were discussed until consensus was reached.

### Ethical considerations

Ethical approval for the study was granted by the Research Ethical Committee of the National Taiwan University Hospital (No. 200809056R). Each participant was informed of the purpose and design of the study and assured that participation was voluntary. Caregivers were required to provide signed informed consent prior to their inclusion. Confidentiality and anonymity were also ensured.

### Findings

Analysis of the interview data identified four core categories that describe the family caregivers’ experiences of managing incontinence: chaos, hypervigilance, exhaustion, and creating a new life. Nine related subcategories further describe their experiences: fluster, dirtiness, urgency, fear of potential health-hazard, physically demanding and time-consuming, mentally draining, financial burden, learning by doing, and attitude adjustment (Table 2).

**Table 2:** The overall theme and subthemes that emerged from the interview.

Themes	Subthemes
**Chaos**	Fluster
	Dirtiness
**Hypervigilance**	Urgency
	Fear of potential health-hazard
**Exhaustion**	Physically demanding and time-consuming
	Mental draining
	Financial burden
**Creating a New Life**	Learning by doing
	Attitude adjustment

**Fig 1:**
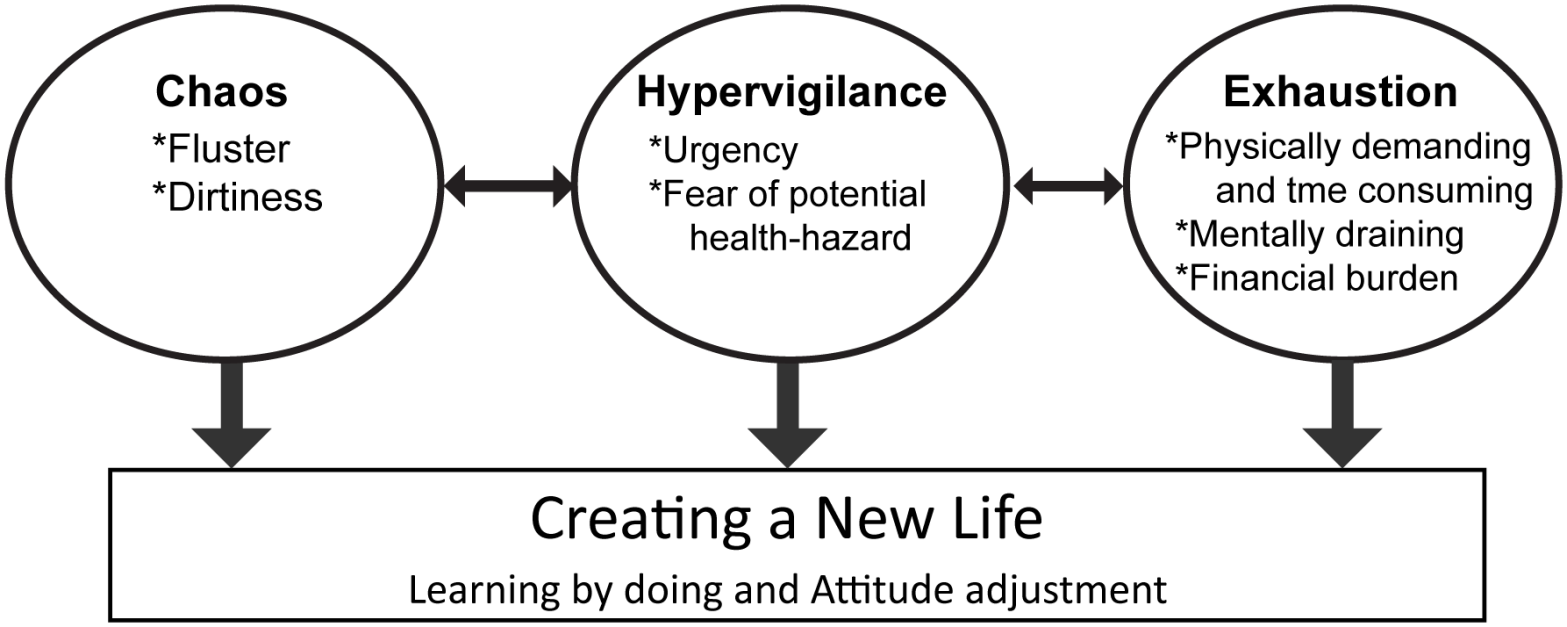
The categories and subcategories that emerged from the interviews.

Taken together, these describe a process of struggling to cope with the care of stroke survivors with urinary or fecal incontinence. Of the four main categories, “creating a new life” appeared to develop gradually over time in an attempt to orient caregivers to their new life, while the other three categories were present continuously and seemed interminable (Fig 1).

### Category 1: Chaos

Chaos describes the absence of order or the confusion that resulted from caring for the stroke survivor’s incontinence, compared to the caregiver’s previous life. The care of incontinent patients requires significant levels of attention and concern. Inexperienced family caregivers initially face chaos due to feeling flustered by the unpredictability of incontinence and the dirtiness that requires a significant amount of cleaning. Caregivers need to overcome the chaos, so that they may go a step forward to adopt other strategies for coping with further challenges without the concerns of being contaminated, disturbed, or anxious.

#### Subcategory: Fluster

Fluster is a feeling nervousness or pressure due to multiple skills and procedures needed, including changing positions safely, cleaning, and changing diapers. Caregivers often felt it was hard to get used to all of these tasks, which caused additional problems. For example, they must not only change a diaper but may also need to clean bed sheets, floors, and even the whole room. More tasks led to greater levels of fluster. For example, one son-in-law said, “*I thought that I should wear the gloves but it just happened so fast… I was flustered and wanted to clean up all the mess as soon as possible*”.

A son noted, “*Changing diapers sounds easy*, *but anything may happen*. *I was always nervous and flustered whenever an unexpected situation happened when dealing with incontinence*”.

One wife described her situation by stating, “*I have to lift him up while wiping and doing lots of tasks at the same time…sometimes feces spreads … and we even change the bed sheets*”. Furthermore, these tasks are often repeated again and again. One daughter said, “*The stool is uncontrollable; it came out just after you thought it’s over*”.

#### Subcategory: Dirtiness

The unpleasant smell of feces and urine, and incomplete cleaning that can leave the environment feeling dirty, disgusting, and uncomfortable was the subcategory of dirtiness. For example, one son-in-law responded, “*The uncontrollable pee and poo make the room smell bad and uncomfortable…dirtiness makes me feel upset*”. Another daughter mentioned, “*I felt dirty in the beginning … I did find it disgusting… If you do not change in time*, *the urine will spread all over the bed… the room will smell bad and dirty and it’s hard to bear*”.

When caregivers had no knowledge of how to provide care and deal with incontinence and solve unexpected problems, they experienced fluster and anxiety.

### Category 2: Hypervigilance

Caregivers not only have to overcome the concerns of dirtiness and fluster, but must also pay attention to the feelings and health of the stroke survivors. Therefore, caregivers must remain alert, so that they can act immediately and keep the stroke patients clean and comfortable. Moreover, being alert helps caregivers avoid unnecessary care work, such as washing pants, sheets, blankets, and floors, which can prevent potential complications such as bedsores or infection.

#### Subcategory: Urgency

It is imperative that incidences of incontinence be cared for immediately; urgent intervention is required and cannot be delayed. If caregivers cannot provide immediate care, the patient is left in an uncomfortable state. Therefore, urgency was a factor that necessitated caregivers’ hypervigilance. One son-in-law noted, “*After the stool passage*, *she cannot wait…I should deal with it quickly and leave other things behind…other daily living care could be arranged*, *and followed my schedule*, *but not incontinence…*”. A granddaughter responded, “*She did not know (about the stool passage) and tell us…so*, *we should deal with it as soon as possible*, *because the longer you left it there*, *the harder it was to clean it up…*”.

#### Subcategory: Fear of Potential Health-Hazard

The hypervigilance was also driven, in part, from the fear of potential health complications associated with not cleaning fecal incontinence promptly. These included the possibility of an increase in patient discomfort, impaired skin integrity, infections, hospitalizations, medical costs, and caregiver loading, and a decrease in the quality of the caregiver’s life. For example, a wife stated:

*I should change things immediately after he urinates or defecates*, *or else his buttocks will get red and injured… I can feel his pain… If you miss once or twice*, *his skin will be injured and that will be an additional problem you have to deal with…*.

The urgency and fear of the potential health-hazards of urinary and fecal incontinence created increased nervousness and alertness in the family caregivers, and ultimately, a state of constant hypervigilance.

### Category 3: Exhaustion

Exhaustion describes the state of feeling tired-out—physically, mentally, and financially—due to hard caring work. Physical care is a trifling “around the clock” job. It was more difficult for caregivers dealing with both incontinence and physical dependency.

#### Subcategory: Physically Demanding and Time-Consuming

The necessary care tasks for stroke survivors are complicated and physically demanding, particularly for caregivers that are not professionally trained. Consequently, the physically demanding care that is required was cause for caregivers’ experience of exhaustion when managing incontinence care. One wife described her experience by stating:

*… for stool cleaning and diaper changing… I feel soreness and numbness of my hands at night*. *I had back pain and I get more frequent headaches now…I wake up several times a night to check his diaper and don’t get enough sleep…It’s tiring*.

One son said, *“Although I am a male*, *changing diapers consume lots of energy*. *Every time I finish the diaper changing I feel my back pain…”*.

The arduous management of incontinence is also time-consuming, which adds to the exhaustion. Patients must use the toilet several times throughout the day and night, leaving family caregivers with little time for themselves. A granddaughter observed, “*In addition to diaper changing*, *I also need to wash his pants*, *clean his body*, *and even shower him*. *There are so many things waiting to be done after his urination or stool passage*”.

Caregivers experienced physical problems, such as sleep deprivation, back pain, headaches, and fatigue that led to physical exhaustion, which was compounded by the time-consuming nature of the care and subsequent activities.

#### Subcategory: Mentally Draining

Constant worrying about the patient’s condition caused caregivers to experience mental stress. Common problems were fear of being unable to offer good care, feeling frustrated, and depressed. One daughter mentioned, “*I’m worried about not being able to provide proper care for her*, *and whether she will have complications or infections…I have to offer both money and energy and I’m mentally fatigued…*”. A daughter-in-law also responded, “*I feel pressured and worry about whether I can handle this or not*, *whether she will be all right under my care or not…The invisible stress is huge…*”.

#### Subcategory: Financial Burden

Management of incontinence is expensive, resulting from the costs of nutrient supplements, diapers, pads, and other supplies, and adds an additional burden to family caregivers. A son-in-law noted, *“Diapers are more costly because I change them whenever she urinates or defecates … for her good*, *I cannot save this money… I worry about money*. *These are really huge costs…*”. Another daughter said, “*We now feed her the special milk designed for lactose intolerance to prevent diarrhea*. *It costs us more*, *and we have to buy one can every three days*. *This is really costly*”.

Stroke survivor’s physical disability requires a substantial amount of assistance from caregivers on several levels. Incontinence was associated with a lack of control, repetition, and physical dysfunction that contributed to physical demands, consumption of time, mental stress, and financial cost among caregivers.

### Category 4: Creating a new life

Most caregivers needed to learn how to perform each care task, and sought professional assistance for instructions. To handle incontinence, caregivers not only tried to accumulate experience by doing, trying to complete every step smoothly, but also needed to change their perceptions toward excrement by adjusting their attitude and thinking positively. All of these changes meant creating a new life for themselves.

#### Subcategory: Learning by Doing

Caregivers tried to learn the required skills from professionals or other sources, such as books and people with similar experiences. One daughter engaged in problem solving activities, noting,

*My daughter-in-law had experience of caring for elders and kids … I learned how to deal with urination and defecation from her … Besides*, *I read and ensure that food is rich in fiber*, *which affects bowel movement*. *Now she defecates regularly and smoothly*.

One son said,

*In the beginning*, *I was a novice and knew nothing at all*. *If nurses only tell me and do not show me what to do*, *the help was limited … I learn from doing*, *because only doing can make me understand the process … I learned how to turn him and change his diapers … I learned from nurses and accumulated my experience gradually*. *I had to discuss with nurses to understand the reasons*, *and to learn skills for saving time and energy or making him more comfortable…*

Over time, caregivers learned to become experienced and familiar with caring tasks. This made them confident and flexible when meeting different incontinence-related conditions. A daughter-in-law said, “*The first time*, *I may be new to the skills*, *but I’m more sophisticated after a few times of practice*. *Now I have few problems dealing with her*”. A daughter responded, “*…I learned (to) handle it…Now I’m good at this and I feel the situation is not so hard anymore… I will no longer get poo everywhere … I do it smoothly*”.

#### Subcategory: Attitude Adjustment

Caregivers must overcome the feelings of discomfort when providing incontinent care. They must change how they perceive excrement, which is often associated with dirt and bad smells. They must therefore learn to clean the mess quickly without thinking about the dirtiness. A daughter-in-law mentioned “*If I am too rushed to get gloves*, *I just think that we are family*, *so it doesn’t matter… I can wash later*. *It’s all right and I am used to this situation … I do not feel flustered or dirtiness anymore*”. One wife also described a similar feeling “*Now I only worry about whether he can be cured or not*, *and not about whether it’s dirty or not*”.

Besides seeking external assistance, caregivers also adjusted their attitudes to move on and appreciate life more. A son-in-law said,

*There is pressure*, *indeed*, *from taking care of her*. *But I think that this is just the way that life is now*. *All I can do is give as much as I can*, *so that I can say I did my best*”. A wife also remarked “*We should change … and try not to … think of sad and painful situations*. *We should look at the bright side of everything because that’s the life we’re in*. *We cannot change it*, *so the only way (forward) is to accept it and move on*.

Caregivers prepared themselves to overcome their challenges and tried to change their thinking to maintain mental balance. Their goal was to create a new life with acceptable conditions.

## Discussion

This study highlights the unique experiences of family caregivers of stroke survivors with incontinence. It was designed to provide a deeper understanding these caregivers’ experiences and their challenges. We identified four categories that reflected the progress of physical and mental reactions to the care of stroke survivors with incontinence. These were chaos, hypervigilance, exhaustion, and creating a new life. These categories revealed a mix of different feelings and emotions among family caregivers when dealing with incontinence.

Our results are consistent with several previous qualitative studies revealing that family caregivers of patients with strokes experienced hypervigilance [22–24] and exhaustion [4, 25]. Indeed, strokes cause significant functional limitations that required long-term support, making exhaustion and hypervigilance common among caregivers. The daily repetition, time-consuming nature, and physical burden further exacerbate this situation. Although previous studies have reported that family caregivers experienced shame when dealing with incontinence [5] and embarrassment when it happened publicly [22], these were not mentioned in this study. This difference may be due to the sense of responsibility derived from filial obligation and affection in Eastern countries, with family caregivers considering that the incontinence was caused by the stroke, not by the patient [26]. Assisting with personal care needs is a debt of gratitude for the care the parent provided when the caregiver was young. Another possibility is that hypervigilance may lead to diapers being checked more often, thereby helping caregivers avoid unnecessary additional care work. This may result in less public incontinence, making the issue less pertinent.

Caring for stroke survivors with incontinence is physically demanding, mentally draining, and financially burdensome to family caregivers. Almost all caregivers mentioned drains on their mental health, personal time, physical health, and financial resources, particularly the latter two. The physical aspects of incontinence care of elderly stroke survivors with immobility, and their greater dependence and 24-hour care requirements, led to back pain, sleep deprivation, and fatigue. This suggests that family caregivers may require, and benefit from, respite care, consistent with the finding of previous studies exploring the needs of stroke caregivers [4, 27]. We propose that healthcare providers provide information about the availability of respite care to ensure family caregivers gain appropriate assistance in sharing their workload; in turn, this could help avoid unnecessary institutionalization of stroke survivors.

Financially, the extra expense of incontinence care is high, such as nutrient supplements (special formulas to prevent diarrhea), diapers, pads, hospitalizations, and emergency visits for incontinence-related complications. Many family caregivers commented on the financial impact of caring for stroke survivors with incontinence. As reported by Cassell and Watt, family caregivers require financial support from the government [22]. In many instances however, government funding programs and the social welfare system could have funded much of the equipment purchased by caregivers, if they had known about them. This lack of awareness of resources was aptly described when one family caregiver observed, “*I do not have enough time to sleep; how could I have the energy to understand the complicated application process for social welfare*”. Hence, we suggest that the government not only offer sufficient allowances for family caregivers but also ensure adequate information is publically available about the application process and available support. The use of pamphlets or the mass media could improve access and reduce caregiver burdens, thereby increasing the effectiveness of the social welfare system.

In this study, caregivers reported using various strategies to deal with incontinence, despite lacking the knowledge and skills needed to provide incontinence care. The categories of chaos, hypervigilance, and exhaustion were associated with this lack of information, knowledge, and skills about how to deal with incontinence. Caregivers are inexperienced and often insufficiently prepared to take on such responsibilities. Our findings indicated the lack of well-organized training and educational programs, as well as the absence of ongoing support, left the caregivers vulnerable. Simultaneously, family caregivers also seemed to need access to professionals that could be resources of information and skill building. As noted in other studies [22, 25, 28], caregivers require education for daily practical care. However, in the absence of adequate information regarding incontinence care from medical professionals, we found that caregivers were often able to acquire the required knowledge and skills from other resources. It is important for healthcare professionals to provide the knowledge and skills necessary for caregivers to deal with incontinence.

In addition to feeling high levels of burden, caregivers described how they tried to deal with patient’s incontinence problem by learning and making attitude adjustments to create a new life. Specifically, they often tried to develop more positive attitudes towards their situation. Family caregivers needed to overcome the sense of chaos and adopt strategies to cope with their challenges without the concerns of being contaminated, disturbed, or anxious. This experience was confirmed in a recent study [4] in which female caregivers of stroke survivors reported “creating a new normal” and “adapting to a new reality” was important for accepting their new situation. Caregivers not only described how they did their best to care for patients but also how they adjusted their attitudes to accept the situation and move on. The strategy of learning by doing was important for providing incontinence care, and planning and preparation ultimately helped caregivers provide incontinence care more efficiently. Attitude adjustment then enabled caregivers to accept and appreciate life.

### Limitations and suggestions

Despite its contributions, this study had some limitations. The caregivers in this study were recruited from one medical center, and the sample size was small. The research focused only on the incontinence aspect of stroke for family caregivers in Taiwan. Thus, the results may not be transferred to other settings or caregiver populations. The family caregivers were asked to recall their experiences of caring for close family member survivors with incontinence, which may be influenced by recall bias. Furthermore, the caregivers interviewed in this study were not followed, and the differences in incontinence care could not be further described. We suggest that prospective studies be conducted, utilizing both qualitative and quantitative research designs, to understand changes of caring experience across different stages. Extending the scope of investigation to the impact on other issues such as changes in familial relationships and careers for caregivers could provide deeper understanding of care-giving phenomena.

## Conclusion

This study focused on the unique caring experiences of family caregivers of elderly stroke survivors with incontinence. Family caregivers described reactions to urinary/fecal incontinence as a sense of chaos, hypervigilance, and exhaustion, and of the need to create a new life. Using various resources, family caregivers were able to develop the skills and techniques required to deal with the complexities of stroke and incontinence. In most cases, learning was through experience. Moreover, adjusting their attitudes to be more positive and forward-looking helped caregivers bridge their difficulties and continue providing care. Caregivers were eager to gain technical support from healthcare professionals and financial support from the government. Recognizing the physical and emotional reactions of family caregivers caring for stroke survivors with incontinence may help nurses provide better support and resources to meet the needs of caregivers and patients alike.
